# Epigenetic modification with trichostatin A does not correct specific errors of somatic cell nuclear transfer at the transcriptomic level; highlighting the non-random nature of oocyte-mediated reprogramming errors

**DOI:** 10.1186/s12864-015-2264-z

**Published:** 2016-01-04

**Authors:** Sayyed Morteza Hosseini, Isabelle Dufort, Julie Nieminen, Fariba Moulavi, Hamid Reza Ghanaei, Mahdi Hajian, Farnoosh Jafarpour, Mohsen Forouzanfar, Hamid Gourbai, Abdol Hossein Shahverdi, Mohammad Hossein Nasr-Esfahani, Marc-André Sirard

**Affiliations:** Department of Reproduction and Development, Reproductive Biomedicine Centre, Royan Institute for Biotechnology, ACECR, Isfahan, Iran; Department of Genetics, Reproductive Biomedicine Research Center, Royan Institute for Reproductive Biomedicine, ACECR, Tehran, Iran; Centre de Recherche en Biologie de la Reproduction, Faculté des Sciences de l’Agriculture et de l’Alimentation, Département des Sciences Animales, Pavillon INAF, Université Laval, Québec, QC G1V 0A6 Canada

**Keywords:** Somatic cell nuclear transfer, Trichostatin A, Transcriptome, Bovine

## Abstract

**Background:**

The limited duration and compromised efficiency of oocyte-mediated reprogramming, which occurs during the early hours following somatic cell nuclear transfer (SCNT), may significantly interfere with epigenetic reprogramming, contributing to the high incidence of ill/fatal transcriptional phenotypes and physiological anomalies occurring later during pre- and post-implantation events. A potent histone deacetylase inhibitor, trichostatin A (TSA), was used to understand the effects of assisted epigenetic modifications on transcriptional profiles of SCNT blastocysts and to identify specific or categories of genes affected.

**Results:**

TSA improved the yield and quality of in vitro embryo development compared to control (CTR-NT). Significance analysis of microarray results revealed that of 37,238 targeted gene transcripts represented on the microarray slide, a relatively small number of genes were differentially expressed in CTR-NT (1592 = 4.3 %) and TSA-NT (1907 = 5.1 %) compared to IVF embryos. For both SCNT groups, the majority of downregulated and more than half of upregulated genes were common and as much as 15 % of all deregulated transcripts were located on chromosome X. Correspondence analysis clustered CTR-NT and IVF transcriptomes close together regardless of the embryo production method, whereas TSA changed SCNT transcriptome to a very clearly separated cluster. Ontological classification of deregulated genes using IPA uncovered a variety of functional categories similarly affected in both SCNT groups with a preponderance of genes required for biological processes. Examination of genes involved in different canonical pathways revealed that the WNT and FGF pathways were similarly affected in both SCNT groups. Although TSA markedly changed epigenetic reprogramming of donor cells (DNA-methylation, H3K9 acetylation), reconstituted oocytes (5mC, 5hmC), and blastocysts (DNA-methylation, H3K9 acetylation), these changes did not recapitulate parallel marked changes in chromatin remodeling, and nascent mRNA and OCT4-EGFP expression of TSA-NT vs. CRT-NT embryos.

**Conclusions:**

The results obtained suggest that despite the extensive reprogramming of donor cells that occurred by the blastocyst stage, SCNT-specific errors are of a non-random nature in bovine and are not responsive to epigenetic modifications by TSA.

**Electronic supplementary material:**

The online version of this article (doi:10.1186/s12864-015-2264-z) contains supplementary material, which is available to authorized users.

## Background

Early embryonic development in mammals begins in transcriptional silence [1] with an oocyte-mediated transcriptional reprogramming of parental gametes occurring during an across-the-board process of “erase-and-rebuild” [2]. In this process, the parental transcription programs are erased long before (maternal) or soon after (paternal) fertilization to generate a relatively naïve zygotic chromatin upon which the transcription program of a new life cycle is rebuilt de novo after activation of the zygotic genome [2, 3]. Any perturbation in this process will result in ill or fatal transcriptional phenotypes of the resultant embryos [4]. The very few viable clones obtained at the end of a typical cloning experiment underscore that substantial differences exist between transcriptional reprogramming of somatic cell nuclei and gametes [5].

Six microarray studies have been carried out to explore the transcriptomic profiles of cloned (SCNT) vs. fertilized (IVF) bovine blastocysts [4, 6–10] and all reported extensive transcriptional reprogramming of the donor cells by the blastocyst stage with few genes, even as low as twenty [8], being deregulated in SCNT embryos. Conversely, numerous studies have reported abnormal patterns of DNA-methylation and histone acetylation/methylation of bovine clones [11–14]. These studies concluded that bovine SCNT embryos suffer from genome-wide hypermethylation, associated with elevated heterochromatic histone methylation (H3K9me2) and H3K9 acetylation in the trophectoderm layer. Therefore, a typical SCNT embryo may be transcriptionally close to (euchromatin), but epigenetically far from (heterochromatin), normal embryos, demonstrating that epigenetic but not expression barriers limit reprogramming efficiency [15]. Accordingly, it would be interesting to determine how small transcriptional aberrations translate into broad epigenetic anomalies or vice versa.

Somatic cell chromatin is compact and static, due to the tight association of chromatin with heterochromatin binding proteins and of histones with chromatin. Proper erasure of these epigenotype markers is essential for chromatin remodeling and pluripotency, as restricting the exchange of these chromatin factors makes the chromatin inaccessible to oocyte reprogramming factors [13]. The short duration and compromised efficiency of the erasure process is considered to be the main cause of the transcriptomic and epigenomic anomalies of cloned embryos [16]. To circumvent this problem, assisted epigenetic modification of somatic cells, before and/or soon after SCNT, with DNA-methyltransferase (DNMTi) and histone deacetylase (HDACi) inhibitors has been recently used to assist the endogenous epigenome-modifying machinery and enhance the speed and extent of nuclear reprogramming (for review see [17]). Among these, members of the hydroxamic acid-containing class of HDACi such as Trichostatin A (TSA) and Scriptaid are used more frequently due to their potent effects on global acetylation. Given the genome-wide effects of HDACi, increased acetylation results in the increased chromatin relaxation at locations that are associated with pluripotency, and this is expected to provide a chromatin conformation more permissive to transcriptional activation [18].

Treatment with TSA improved the development of SCNT bovine [17–19] and pig [20, 21] embryos to the blastocyst stage. In mice, TSA improved both nuclear remodeling [22] and development to term [23–25]. Treatment with TSA also induced a parallel increase in histone acetylation, chromosome decondensation and nuclear volume in SCNT mouse embryos similar to that observed in ICSI-derived embryos [26]. Importantly, through analysis of chromatin remodeling and 5-bromouridine 5′-triphosphate (BrUTP)-labeled RNA, Bui et al. [26] and VanThuan et al. [27] demonstrated a more effective formation of DNA replication complexes in TSA and Scriptaid treated embryos which was evident by enhanced levels of newly synthesized RNA and a significant reduction in asymmetric expression of nascent RNA. Notably, a positive correlation between the increase in nascent RNA synthesis and full-term development of cloned mice was observed. In cloned pig embryos, Whitworth et al. [28] reported that Scriptaid treatment significantly affected the expression of 7 of the 14 transcripts evaluated and returned three of them to levels observed in in vivo-derived embryos. Even though a single defect in transcription reprogramming may cause a “ripple” effect resulting in the aberrant expression of entire networks of downstream target genes [29], the analysis of a small number of transcripts is of limited value for the systematic study of the genetic interactions involved in a complex trait. Post genomic era approaches, including genome wide analyses and network investigations, are needed.

This is the first study that seeks to assess the effects of assisted epigenetic modifications on transcriptional profiles of bovine SCNT embryos with a complete embryo RNAseq-derived microarray which includes splice and 3′UTR variants [30]. To investigate the potential meaning of the alteration of specific genes or categories of genes, we also attempted to associate specific mechanisms or events that occur in somatic cells, reconstituted oocytes and developing embryos with the observed alterations. Since no study has examined the effects of HDAC inhibitors on transcriptome and associated events, our results will expand the knowledge in this enigmatic field.

## Results

### TSA treatment improves in vitro, but not in vivo, developmental competence of SCNT bovine embryos

The custom *in vitro* measure of cloning efficiency is the yield and quality of blastocyst formation [5]. In our results (Additional file 1: Table S1A), cleavage rate was not significantly different between the groups. However, TSA treatment boosted blastocyst development to a rate that was significantly higher than CTR-NT, but not IVF (39.8 ± 4.1 vs. 28.7 ± 5.5 vs. 34.3 ± 3.9 %, respectively). Development to grade 1 & 2 blastocyst was not different between TSA-NT and IVF (48.0 ± 6.0 vs. 41.7 ± 4.2 %, respectively) but significantly less in CTR-NT (34.7 ± 6.7 %) compared to TSA-NT. Differential staining of blastocysts (Additional file 1: Table S1B) revealed a comparable total cell number between the groups. However, TSA treatment, but not the embryo production method, changed the distribution of cells in blastocysts as the proportion of cells allocated to the ICM and ICM/TCN proportion, were both significantly higher in TSA-NT blastocysts compared to CTR-NT embryos.

The ultimate readout of cloning efficiency is the ability of SCNT embryos to develop into viable offspring [5]. Although numbers are low, the transfer of grade 1 & 2 blastocysts resulted in comparable early (days 35–40) pregnancy rates between the groups (Additional file 1: Table S1C). However, total percentages of pregnancy loss between critical pregnancy days 60–120 were high for both NT groups with no beneficial effect of TSA treatment when compared to no failure in IVF pregnancies. In addition, a number of pregnancies from SCNT in both groups were terminated at days 100–235 due to excessive accumulation of amniotic fluid (hydrops) and other complications (CTR-NT: 33.3 %, TSA-NT: 50.0 %). Elective C-section was performed between days 286 to 290 of pregnancy resulted in the delivery of 6 CTR-NT and 2 TSA-NT calves. Four CTR-NT claves and one TSA-NT calve did not survive. The deceased calves suffered from placental abnormalities including placental hypertrophy and small numbers of larger placentomes.

### TSA treatment, not the SCNT process, is the greatest source of variation at the transcriptome level

**Fig. 1:**
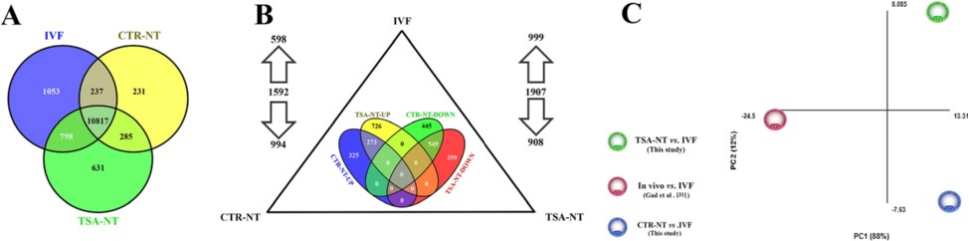
Microarray graphical results. **a** Overview of differential gene expression in CTR-NT and TSA-NT day 7 blastocysts compared to IVF counterparts. The total number of differentially regulated genes which comprised the total number of upregulated and downregulated genes is shown by directions of arrows. The Venn diagram shows the overlap between deregulated genes in both SCNT groups. **b** Venn diagram illustration of the overlap between genes predominantly and exclusively expressed in CTR-NT, TSA-NT and IVF -derived blastocysts. **c** Two-dimensional principal component analysis (PCA) indicating the source of variation in the overall transcriptional profiles of CTR-NT vs. IVF and TSA-NT vs. IVF in this study with the transcriptional profile of in vivo blastocysts obtained from the study of Gad et al. (2012) using the same array platform

Significance analysis of microarray (SAM) of blastocysts revealed that out of the 37,238 targeted gene transcripts represented on the microarray slide, a relatively small number of genes were differentially expressed (DEG) in CTR-NT (1592 = 4.3 %) and TSA-NT (1907 = 5.1 %) embryos compared to IVF (FDR 1.5, P ≤ 0.05, FC ≥1.5) (Fig. 1a). Of the DEGs identified, 598 and 999 genes were upregulated and 994 and 908 genes were downregulated in CTR-NT and TSA-NT embryos, respectively. The identity and description of top molecules and each differentially expressed transcripts in CTR-NT and TSA-NT blastocysts are listed in Additional file 2: Table S2 and Additional file 3: Table S3, respectively. Comparisons between genes commonly upregulated or downregulated between CTR-NT and TSA-NT embryos revealed that approximately almost all of the downregulated genes in either group, half of the upregulated genes in CTR-NT, and one third of the upregulated genes in the TSA-NT group were common. Notably, no upregulated gene in one SCNT group was downregulated in the other group and vice versa (Fig. 1a).

Owing to the similarities between CTR-NT and TSA-NT array results, it was interesting to define the probe sets exclusively expressed in the blastocysts of either group. Out of the 37,238 targeted gene transcripts on the microarray slide, 231, 631, and 1053 probe sets had a signal higher than the summation of background intensity plus two times the SD of background, indicating the unique presence of the mRNA for these genes in CTR-NT, TSA-NT, and IVF embryos, respectively (Fig. 1b and Additional file 4: Table S4). From this list, HDAC11 and FZD7 were expressed in CT-NT; WNT9B, TET2, FZD10, IGF1R, STAT6, and H2AFY2 were expressed in TSA-NT; and XIST, ELK4, FZD3, NOTCH1, PDE3A, KDM2B, and FOXO4 were expressed in IVF embryos.

### TSA does not correct specific transcriptional errors of SCNT in bovine

**Fig. 2:**
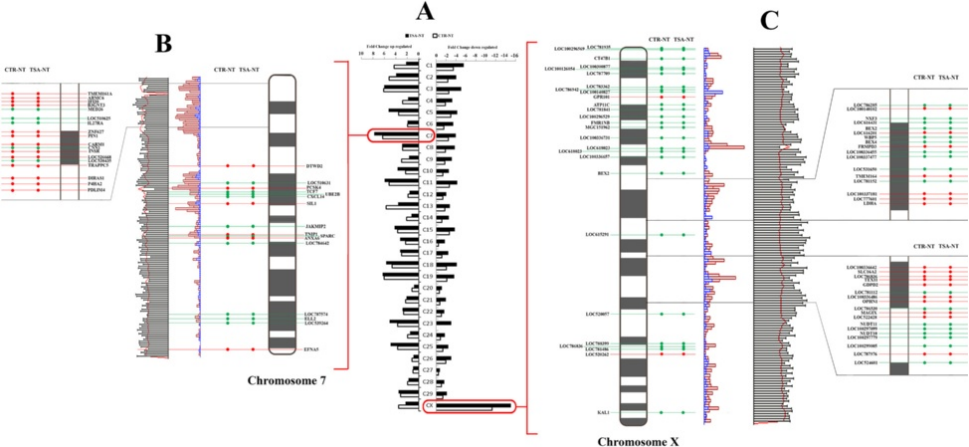
Temporal and spatial map views of DEGs in CTR-NT and TSA-NT blastocysts. **a** Chromosomal distribution of differentially expressed genes in CTR-NT (open bars) and TSA-NT (black bars) -derived blastocysts. **b** & **c** Breakdown of DEGs on chromosome 7 (**b**) and X (**c**) based on their location maps in Ensembl data base (http://asia.ensembl.org). The red and blue graphs represent the number of protein-coding genes and short non-coding genes in area, respectively. The black line graphs represent the number of repeat sequences and the red graphs represent GC percentages. The red and green circles represent upregulated and downregulated genes, respectively, in CTR-NT and TSA-NT-blastocysts. The magnified panels are to better show the genes located in these chromosomal regions

To investigate the possibility of reprogramming error hotspots, the chromosomal distribution of deregulated genes was reviewed to identify gene loci preferentially affected in both groups. Chromosomal map view of deregulated genes revealed a high degree of consistency between CTR-NT and TSA-NT blastocysts (Fig. 2a). The upregulated genes were evenly distributed while chromosome 7 (Fig. 2b) contained a higher number of deregulated genes. A bias in the distribution of downregulated genes was observed with as many as 15 % of the transcripts in both SCNT groups from genes located on chromosome X (CTR-NT: 147/994, TSA-NT: 136/906) (Fig. 2c).

**Fig. 3:**
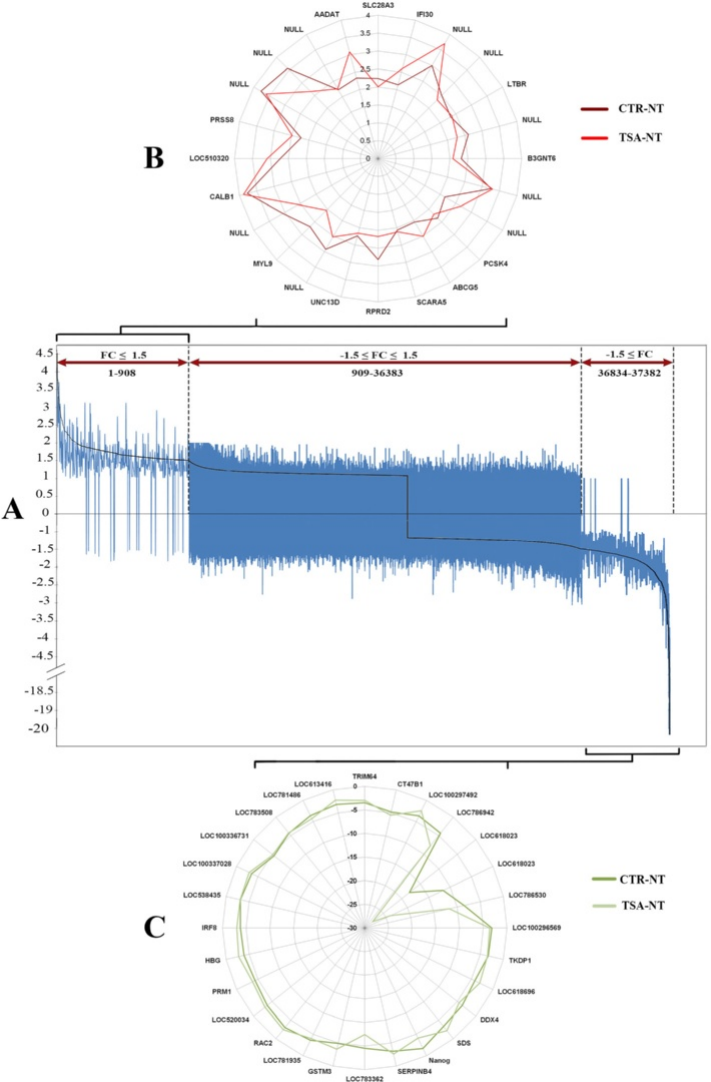
The convergence and divergence between CTR-NT and TSA-NT transcriptomes. **a** Direct comparison between total array results of both SCNT groups. All genes were identified for direct comparison with reference to CTR-NT array. **b** & **c** Overlaps between top DEGs that were upregulated (**b**) and downregulated (**c**) in CTR-NT and TSA-NT blastocysts

In order to decipher the location and proportion of genes that could be related to this conflict, the whole transcriptome patterns of both groups were viewed for a direct comparison on the same graph (Fig. 3a). Analysis of microarrays using 5 % FDR and at least 1.5 fold changes (FC) revealed that genes with FC ≤ 1.5 and FC ≤ −1.5 had the greatest and lowest similarities between both groups, respectively; whereas genes with −1.5 ≥ FC ≤1.5 (non DEG transcripts) were the source of greatest variation between the two groups. Figure 3b and c represent the overall top DEGs similarly upregulated and downregulated in CTR-NT and TSA-NT blastocysts, respectively.

Even if the TSA-NT blastocysts were transcriptionally different from the IVF blastocysts, our previous study, using the same platform, revealed a great divergence between the transcriptomes of *in vitro*- and in vivo-derived bovine blastocysts [31]. This led us to determine whether the divergence observed between TSA-NT vs. CTR-NT and IVF could be related to the effect of TSA treatment on the correction of genes commonly deregulated in CTR-NT and IVF embryos. Principal component analysis (PCA) comparison between the transcriptomes of CTR-NT vs. IVF, TSA-NT vs. IVF in the present study with in vivo blastocysts (in Gad et al. [31]) clustered the in vivo blastocysts away from the other groups, indicating a divergence between TSA-NT and in vivo blastocysts at the transcriptome level (Fig. 1c). Among the genes that were considered deregulated in CTR-NT and TSA-NT compared to IVF, 131 and 68 upregulated and 16 and 12 downregulated genes had closer expression levels to in vivo embryos, respectively (data not shown).

### WNT and FGF canonical pathways are similarly affected in CTR-NT and TSA-NT blastocysts

**Fig. 4:**
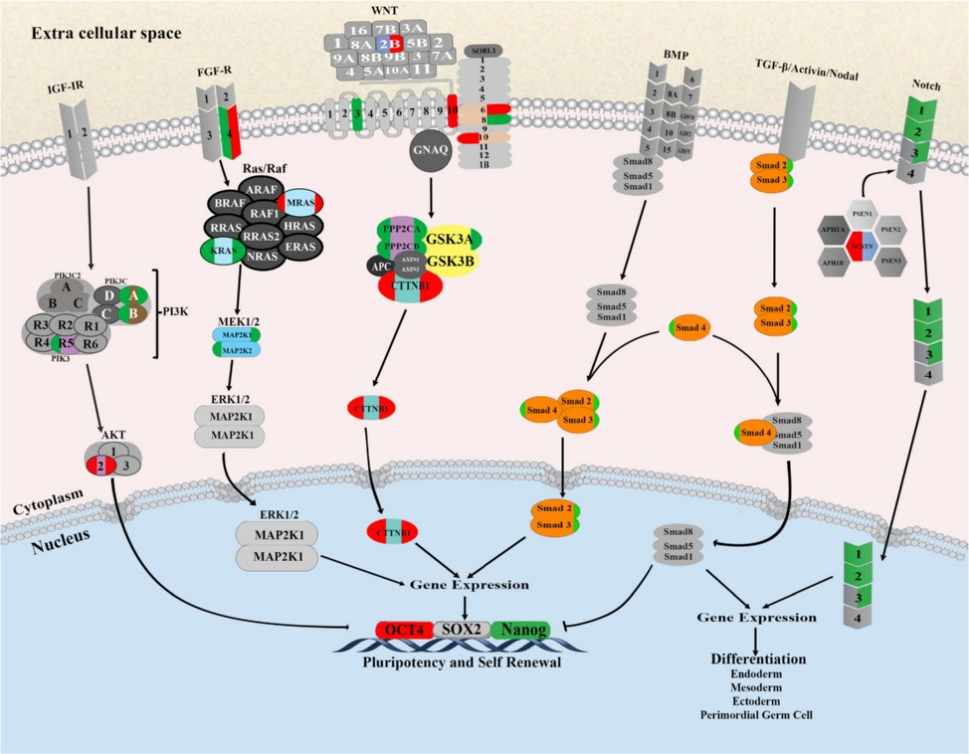
Signaling pathways in CTR-NT and TSA-NT blastocysts. Overview of expression pattern of genes involved in ESC pluripotency and differentiation in the CTR-NT and TSA-NT array results. Each gene can be upregulated, downregulated, or of no-significant expression based on its color in red, green, and gray, respectively. Each gene represents the expression level in both NT groups, the right half of each gene symbol represents expression level in TSA-NT and the left half represents the expression level in CTR-NT embryos

The core pluripotency triad (OCT4, NANOG, and SOX2) was similarly affected in both NT groups: OCT4 was upregulated, NANOG was downregulated, and SOX2 expression was similarly expressed compared to IVF. Treatment with TSA did not change the expression of OCT4 and SOX2 and resulted in further downregulation of NANOG (4-fold compared to the 1.7-fold decrease that was observed in CTR-NT compared to IVF). A reference diagram showing the interactions of five canonical pathways with OCT4, SOX2 and NANOG (http://www.cellsignal.com/) was used to extrapolate the possible pathways that could lead to the observed expression patterns of the genes of the core pluripotency triad (Fig. 4). Examination of genes involved in different canonical pathways revealed that the transcripts for the cytoplasmic mediators of the FGF pathway: PI3K, MEK1/2, were not functionally expressed in the SCNT groups; whereas a few isotypes of the nuclear mediators AKT, ERK1/2, were upregulated. WNT appeared not functional at upstream regulators and destructive complexes of β-CATENIN: PP2A, AXIN, GSK3, APC, were not in an active state to trigger β-CATENIN cytoplasmic ubiquitination, and correspondingly, β-CATENIN transcripts were apparently free to be shuttled into the nucleus in an upregulated form. TGF-β and BMP were in a repressed state as their upstream and downstream regulators were almost downregulated or not significantly expressed compared to IVF. Notably, a bias was observed in the expression of SMADs between the NT groups: SMAD2 and 3 were both downregulated in CTR-NT while SMAD4 was downregulated in TSA-NT. The expression pattern of the NOTCH signaling pathway was similar between the groups with the exception of NOTCH3 which was downregulated in CTR-NT but not in TSA-NT blastocysts.

### TSA effects on epigenetic alterations do not recapitulate changes in chromatin remodeling, epigenetic gene expression, and OCT4 and nascent mRNA expression

The heart of cellular reprogramming is epigenetic modifications and it has been suggested that epigenetic modifiers such as TSA have a carryover effect on a wide variety of epigenetic cellular characteristics [26]. To investigate the potential meaning of the alteration of the expression of specific genes or categories of genes, we attempted to determine whether these alterations in gene expression were associated with specific mechanisms or events that occur in somatic cells, reconstituted oocytes and developing embryos.

#### Epigenetic marks

**Fig. 5:**
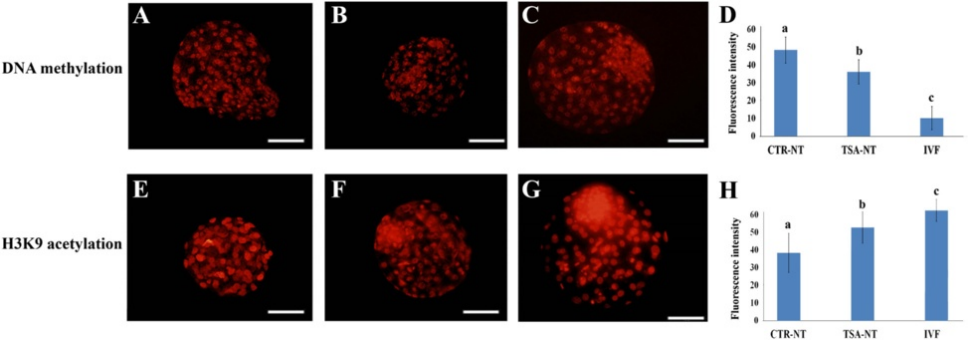
**a**-**h** Immunofluorescence of DNA methylation (**a**-**d**) and H3K9 acetylation (**e**-**h**) in blastocysts developed in CTR-NT, TSA-NT and IVF experiments, respectively. Bars with different letters differ significantly at *P* < 0.05. Scale bar represents 100 μm

Flow cytometry analysis revealed that the TSA treatment significantly increased H3K9 acetylation (280.4 ± 4.1 vs. 128.2 ± 6.3) but had no measurable effect on DNA-methylation (140.6 ± 1.2 vs. 138.1 ± 3.5) in fibroblasts compared to untreated cells. Epifluorescent microscopy analysis of blastocysts (Fig. 5a-h) detected the highest and lowest levels of DNA-methylation in CTR-NT and IVF embryos (48.5 ± 7.3 and 10.3 ± 6.5, respectively). Although TSA treatment significantly decreased DNA-methylation (36.3 ± 6.9) compared to CTR-NT, this level of DNA-methylation was still significantly higher than in IVF embryos. The mean intensity of H3K9 acetylation in IVF embryos was significantly higher than in both NT groups and that of TSA-NT significantly higher than CTR-NT (65.3 ± 6.5 vs. 55.2 ± 9.3 vs. 40.1 ± 11.6, respectively).

**Fig. 6:**
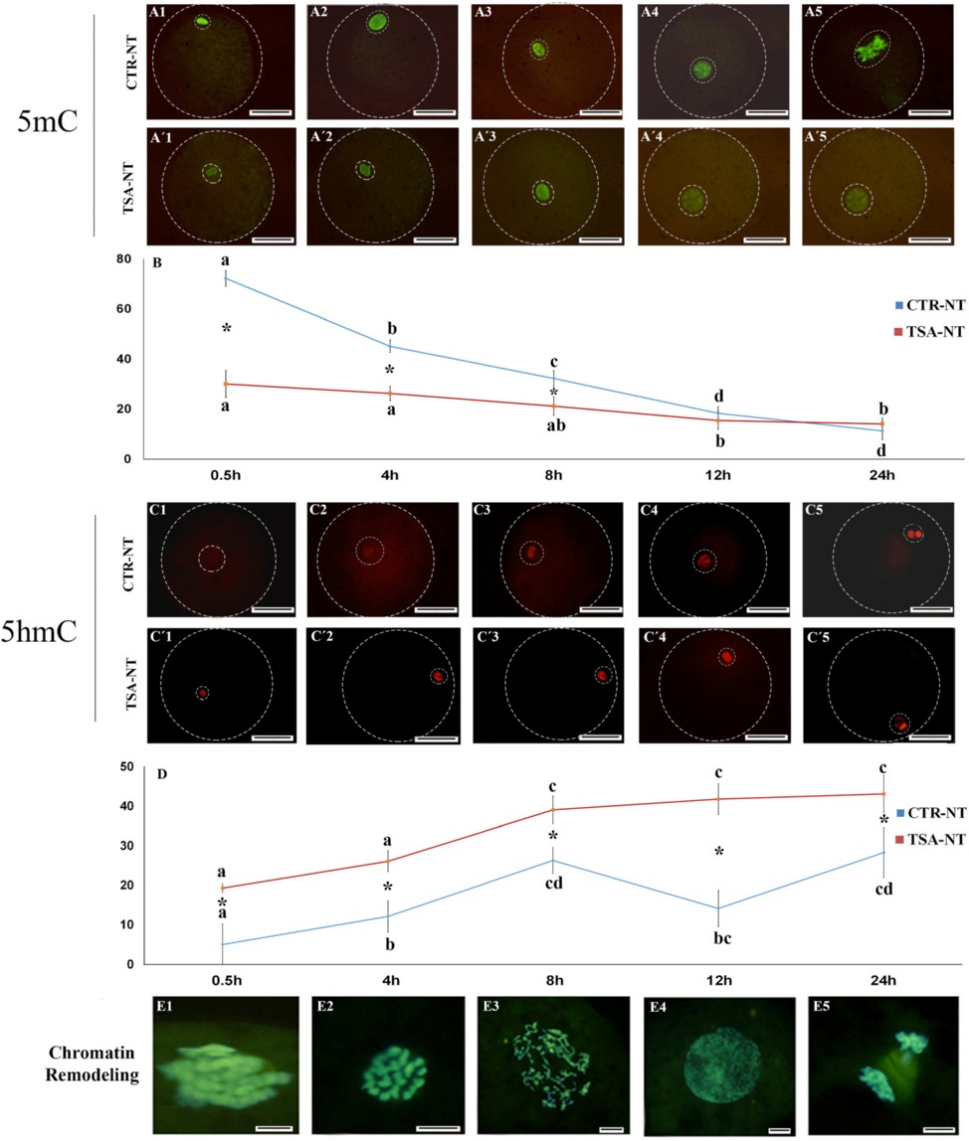
Epigenetic reprogramming and chromatin remodeling in reconstituted oocytes. **a1**-**a’5** Stepwise pattern of 5mC at different intervals (0, 4, 8, 12, 24 hpr) in CTR-NT (**a1**-**a5**) and TSA-NT (**a’1**-**a’5**). **b** Comparison between intensity levels of 5mC at different intervals (0, 4, 8, 12, 24 hpr) in CTR-NT (blue line) and TSA-NT (red line). **c1-c’1** Stepwise pattern of 5hmC at different intervals (0, 4, 8, 12, 24 hpr) in CTR-NT (**c1**-**c5**) and TSA-NT (**c’1**-**c’5**). **d** Comparison between intensity levels of 5hmC at different intervals (0, 4, 8, 12, 24 hpr) in CTR-NT (blue line) and TSA-NT (red line). **e1**-**e5** Stepwise pattern of nuclear remodeling pictured from 0.5 to 24 h post reconstitution. The merged images of microtubule and nuclear immunostaining have been shown. **e1** the very sign of nuclear swelling without spindle formation, **e2** nuclear remodeling without spindle formation, **e3** premature chromosome condensation with developing spindles, **e4** single pronucleus completely developed with an early SCNT zygote,) first mitotic division of the newly developed SCNT zygote. Bars with different letters differ significantly at *P* < 0.05. Scale bar represents 100 μm

Immunofluorescence staining of the reconstituted oocytes showed a marked effect of TSA treatment on the profile of 5mC disappearance and 5hmC generation that is best viewed in Fig. 6 (a1-a’5, b, c1-c’5, d). The peak of 5mC in CTR-NT oocytes was observed immediately following SCNT and a very faint 5hmC was also observed. Subsequently, 5mC gradually disappeared concomitantly with the appearance of 5hmC. The exception was a significant drop of 5hmC at 12 h post-reconstitution (hpr), which corresponds to the time of DNA synthesis [32]. However, TSA-treated fibroblasts contained less than half of the 5mC and approximately 4-fold more of the 5hmC signals compared to what was initially observed in the CTR-NT reconstituted counterparts (30.1 ± 5.4 vs. 72.3 ± 3.1 and 19.3 ± 5.1 vs. 5.1 ± 1.1, respectively). Apart from this initial difference and despite the gradual pattern of 5mC disappearance in TSA-NT oocytes, the 5mC signals that were observed at 24 hpr were very similar between TSA-NT and CTR-NT reconstituted oocytes (14.1 ± 2.4 vs. 11.3 ± 3.6, respectively). The initial higher intensity of 5hmC signals in TSA-NT compared to CTR-NT was also observed at the subsequent stages of assessment.

#### Chromatin remodeling

The kinetics of nuclear remodeling was quite comparable between CTR-NT and TSA-NT reconstituted oocytes (Fig. 6e1-5). At 2 hpr, the majority of the nuclei were still intact with only minor signs of bipolar spindle formation. At 4 hpr, the overall incidence rates of nuclear remodeling were not different between the groups. Irrespective of TSA treatment, the majority of the reconstituted oocytes had prematurely condensed chromosomes on well-structured bipolar spindles. At 12 hpr, the majority of the reconstituted oocytes had a single pronucleus which condensed into two sets of chromosomes in the newly developing blastomeres at 24 hpr.

#### Nascent mRNA expression

Neither embryo production method nor the TSA treatment affected nascent mRNA production. Moreover, all the embryos had early signs of nascent mRNA production on day 3 when they had at least 16 cells. From the 16-cell stage onward, clear signs of nascent mRNA production were observed in the embryos of both NT groups (Additional file 5: Figure S1a-e’).

**Fig. 7:**
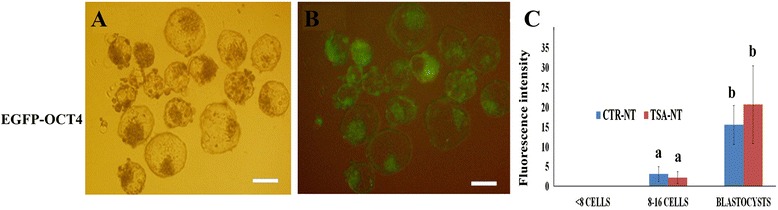
EGFP expression in SCNT embryos harboring EGFP-OCT4 promoter observed under normal light (**a**), and 557 nm excitation of UV filter (**b**). Comparative EGF-Oct4 expression between CRT-NT and TSA-NT embryos at three different stages of in vitro embryo development (**c**). Bars with different letters differ significantly at *P* < 0.05. Scale bar represents 100 μm

Further confirmation of the null effect of TSA on nascent mRNA expression was obtained by using NT embryos reconstituted with fibroblasts carrying the OCT4 promoter-driven enhanced green fluorescent protein (EGFP) (Fig. 7a-c). Irrespective of TSA treatment, EGFP signals, indicative of OCT4 gene activation, were not observed until the embryos reached the morulae stage. All the blastocysts emitted clear EGFP signals (Fig. 7a-c) and the mean intensities of EGFP fluorescence were not significantly different between CTR-NT and TSA-NT blastocysts (25.5 ± 5.6 vs. 22.9 ± 5.2, respectively).

#### Epigenetic gene expression

**Fig. 8:**
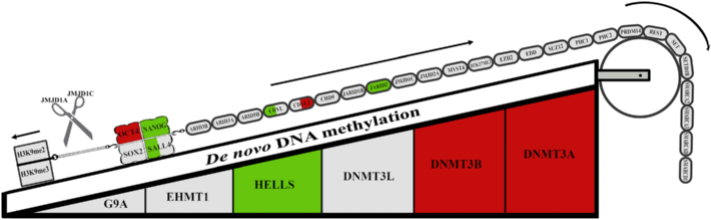
Epigenetic landscape in CTR-NT and TSA-NT blastocysts. Overview of the crosstalk between pluripotency factors and epigenetic modifiers in the CTR-NT and TSA-NT array results. Genes located in the chain are ESC safeguard genes, and genes located in the ramp are differentiating factors. JMJD1C and JMJD1A work through inhibiting the H3K9me2&3 which are also differentiation factors. Each gene represents the expression level in both NT groups, the right half represents expression level in TSA-NT and the left half represents the expression level in CTR-NT embryos

Examination of genes involved in epigenetic regulation of pluripotency revealed that among genes involved in *de novo* DNA-methylation, DNMT3 A & B were upregulated and HELLS was downregulated in both NT groups compared to IVF embryos. Among genes involved in safeguarding epigenetic permissiveness of pluripotency, JARID2 was downregulated in both NT groups, CDYL was downregulated in TSA-NT and CDYL2 was upregulated in CTR-NT groups (Fig. 8).

#### Functional classification and pathway analysis

Ontological classification of differentially downregulated genes using IPA generated several biological processes including cell death and survival, cell-to-cell signaling and interaction, cancer, as well as embryo development as the most significant functions similarly affected in CTR-NT and TSA-NT blastocysts (Additional file 6: Table S5 and Additional file 7: Table S6). However, biological processes emerging from differentially upregulated genes were different between CTR-NT and TSA-NT groups. Cellular assembly and organization were the biological functions most significantly affected in CTR-NT blastocysts with upregulation of the highest number of genes. For TSA-NT-derived blastocysts, cell growth and proliferation, carbohydrate metabolism and molecular transport were the biological functions most significantly affected with upregulation of the highest number of genes.

IPA clustered different top canonical pathways for each group with ratios ranging from 3 to 22 % (proportion of DEGs to the total number of genes involved in each pathway) at *P*-value < 0.05. Aryl hydrocarbon receptor signaling and glutathione-mediated detoxification in CTR-NT, and EIF2 signaling and mitochondrial dysfunction in TSA-NT were the most dominant canonical pathways revealed.

**Fig. 9:**
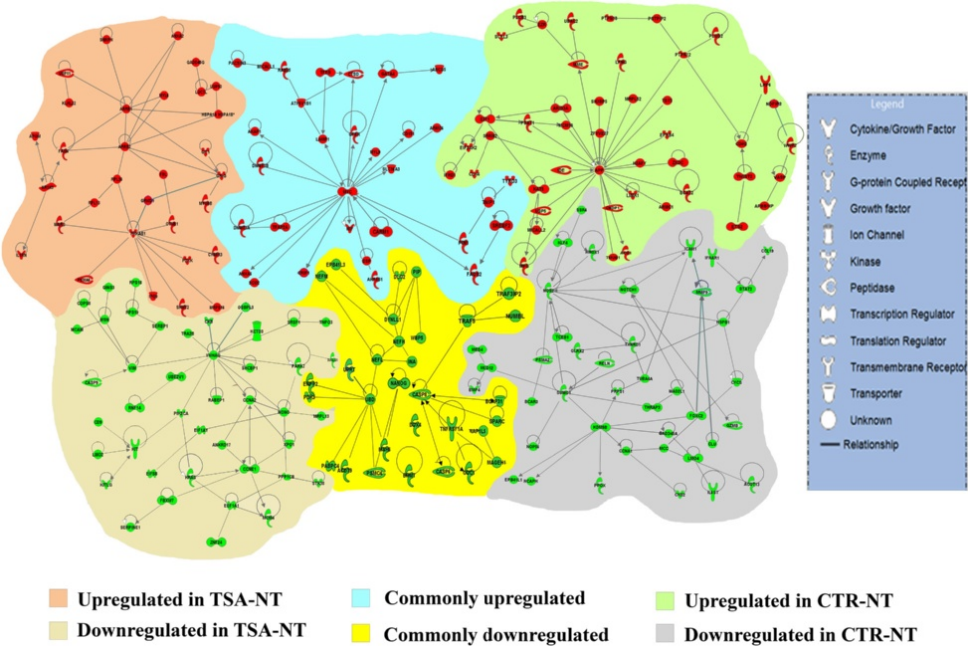
Gene regulatory networks in CTR-NT and TSA-NT blastocysts. Gene regulatory networks representing genes predominantly or commonly up/down -regulated in CTR-NT and TSA-NT -derived blastocysts

The IPA was also queried for the identification of the gene regulatory networks (GRNs) between genes that were either commonly or predominantly expressed in both NT groups (Fig. 9). The MYC-associated regulatory pathway was the dominant pathway that emerged from the genes upregulated in both groups with upregulation of genes involved in epigenetic regulation of pluripotency (DNMT3 A & B, CARM1) and TE lineage (GATA4) [13]. Pluripotency and cell survival pathways were the most stringent pathways that emerged from the genes upregulated in both groups. The central molecules in this pathway were NANOG and CASP8 which act as gate keepers of pluripotency and apoptosis. Among the genes upregulated in CTR-NT vs. IVF, the amyloid precursor protein-associated (APP) pathway emerged with direct relation to TET3, E2F1, ARID3A and FRD1. Several GRN nodes emerged from genes upregulated in the TSA-NT group and this culminated in the upregulation of a number of developmentally important genes including SNRPN, PRKAB1, ARRB2, GADD45G, and GEMIN4. In the same way, several GRN nodes including KDM5B, STAT2, NOTCH1, FOXC2, and HSPB1 were observed within genes upregulated in CTR-NT. Finally, VIM and YWHAG, KIT, HDAC8 were central molecules of the pathways that emerged from genes downregulated in TSA-NT blastocysts.

### Identification of TSA effects on fibroblast gene expression

**Fig. 10:**
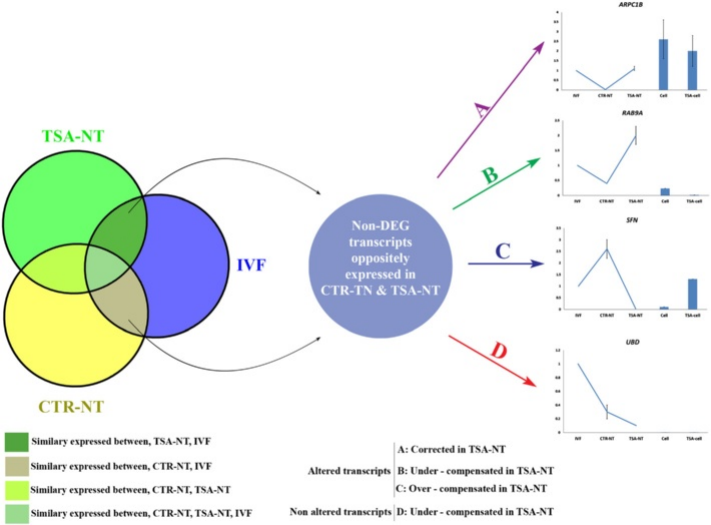
Identification of TSA effects on fibroblast gene expression. Protocol used for identifying effects of TSA on the expression profile of a subset of genes that were oppositely expressed between CTR-NT and TSA-NT blastocysts. The altered transcripts in the fibroblasts that had higher normalized expressions toward IVF in CTR-NT rather than TSA-NT blastocysts were classified as corrected transcripts. The influenced transcripts in the fibroblasts that had higher normalized expressions toward IVF in TSA-NT rather than CTR-NT blastocysts were classified as flawed (over-compensated or under-compensated) transcripts

**Fig. 11:**
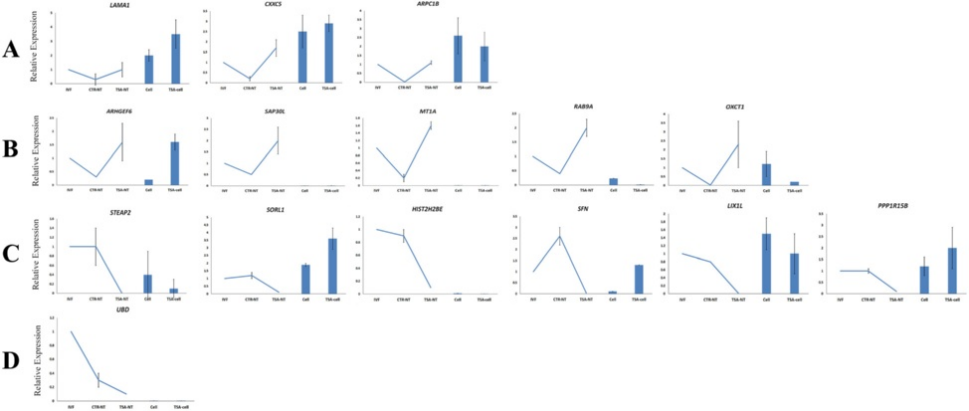
TSA differently affects fibroblast gene expression. Almost transcripts (panels **a**, **b**, and **c**), except for the UBD transcript (panel **d**), were altered after TSA treatment of fibroblasts. Alterations in LAMA1, CXXC5, and ARPC1B resulted in subsequent correction of their expression in TSA-NT blastocyst when compared to IVF. However, alterations in ARHGEF6, SAP30L, MT1A, RAB9A and OXCT1 resulted in subsequent over-compensation of these genes compared to IVF. On the other hand, alterations in STEAP2, HIST2H2B, SFN, SOR1, LIX1L, and PPP1R5B resulted in their subsequent under-compensation in TSA-NT blastocysts

To identify the effects of TSA on fibroblast gene expression, microarray data were queried to select a subset of non-DEG transcripts that were oppositely expressed between CTR-NT and TSA-NT blastocysts. The selected subset of genes included CXXC5, LAMA1, STEAP2, UBD, SAP30L, MT1A, RAB9A, HIST2H2B, SFN, ARPC1B, SOR1, ARHGEF6, LIX1L, PPP1R5B, and OXCT1. Using qRT-PCR, the transcripts that were differentially expressed between treated and untreated fibroblasts were classified as altered transcripts as described by Whitworth et al. [28]. The fibroblast altered transcripts that had higher normalized expression towards IVF in CTR-NT rather than TSA-NT blastocysts were classified as corrected transcripts (Figs. 10 and 11). The fibroblast altered transcripts that had higher normalized expression towards IVF in TSA-NT rather than CTR-NT blastocysts were classified as flawed (over-compensated or under-compensated) transcripts (Fig. 10). In this respect, we observed that all transcripts, except for UBD, were altered after TSA treatment of fibroblasts (Fig. 11). Of these, alterations in LAMA1, CXXC5, and ARPC1B resulted in subsequent correction of their expression in TSA-NT blastocysts when compared to IVF. However, alterations in ARHGEF6, SAP30L, MT1A, RAB9A and OXCT1 resulted in subsequent over-compensation of these genes compared to IVF. On the other hand, alterations in STEAP2, HIST2H2B, SFN, SOR1, LIX1L, and PPP1R5B resulted in their subsequent under-compensation in TSA-NT blastocysts (Fig. 11).

### Microarray validation with qRT-PCR

To validate the microarray results, fourteen genes were selected to include genes that were significantly upregulated (OCT4, CMYC, GATA4), downregulated (XIST, NANOG, GSTM3, CCNB1)) and non-significantly (VEGF, BCL2, SOX2, CDX2, HNF4a, BMPR1B, SMAD1) deregulated according to the microarray results. The overall profiles of gene expression obtained by qRT-PCR revealed positive validation of microarray results as the majority (93 %) of the selected genes had expression profiles consistent with those observed with microarray analysis (Additional file 8: Table S7). The only exception was for the expression of SMAD1. While similar expression levels of SMAD1 were observed with microarray for both NT groups, qRT-PCR showed significant downregulation of SMAD1 in CTR-NT blastocysts. Overall, these validations allow confident interpretation of the results obtained by array hybridization and statistical/functional analysis.

## Discussion

A key characteristic of oocyte-mediated reprogramming to pluripotency is that the process is very inefficient with only a few viable embryos obtained at the end [5]. Etiologically, most studies point towards an aberrant gene expression following errors in the erasure and/or rebuilding of epigenetic marks that can affect totipotency and proper differentiation and development of embryos (for review see [17]). Therefore, several studies have attempted to modify the epigenetic status of donor cells using variants of HDAC inhibitors either before or soon after SCNT. This study represents a collection of physiological, histological and genomic data of unprecedented completeness to understand the complex phenotype of SCNT bovine embryos.

In our study, treatment with TSA was quite beneficial for the *in vitro* development of SCNT embryos compared to control nuclear transfer (CTR-NT) and also IVF embryos. The effect of TSA treatment on *in vitro* development of SCNT embryos is controversial in different species (for review see Monteiro et al. [33]). The initial (epi)genetic background of donor cells may be involved in the generation of different *in vitro* results. For example, while TSA increased cumulus cell-derived cloned mouse blastocysts by 5-fold, it had no effect on the developmental competence of ESC-derived embryos [24]. Similarly, TSA treatment increased blastocyst development of SCNT bovine embryos produced from fetal fibroblasts but not bone marrow cells [21]. Notably, TSA treatment altered the allocation of cells in blastocysts as proportionally more cells were allocated to the ICM of treated embryos, suggesting that an overriding epigenetic modification of the genome before NT has a carryover effect on cell proliferation and differentiation in preimplantation embryos. Ikeda et al. [34] demonstrated a significant increase in ICM number in bovine embryos treated with TSA during IVF. These data convinced us that TSA treatment may provide better prognosis for in vivo development by preparing better quality embryos.

However, post-implantation developmental competence was equally compromised in both groups irrespective of TSA treatment. The majority of NT pregnancies were lost by the middle of gestation and only a few fetuses survived in either group. These results are substantially supported by the fact that even though hundreds of SCNT embryos were transferred since 1996s, the overall success rate in terms of live birth rate has not changed and remains quite low (1–5 %) [35]. Published in vivo studies on HDACis are both limited and controversial: while TSA was quite beneficial in mice [25], it could not improve full-term development in rabbit [36], pig [20], and cow ([37] and the present study). The different cell lines used as well as species/strain-specific differences may be involved in the generation of different in vivo results. For example, Kishigami et al. [24] observed a significant beneficial effect of TSA on the full-term development of B6D2F1, but not of inbred (C57BL/6, C3H/He, DBA/2, and 129/S) mouse clones. Moreover, while reconstituted pig embryos derived from fetal fibroblasts could support full term development irrespective of TSA treatment, bone marrow cells of adult sows could do so only after TSA treatment [21]. This lends support for the different effects of TSA on *in vitro* and in vivo development depending on the nuclear donor cell type. Due to this important difference between *in vitro* and in vivo results of SCNT with TSA treatment, it became relevant to investigate the fundamental molecular pathways involved in pre-implantation SCNT embryo development.

The overall results of transcriptomic analysis indicated that substantial transcriptome reprogramming occurred at the blastocyst stage which is in agreement with all the previous studies in bovine [4, 6–10]. Notably, TSA treatment could not dramatically change the profile of differentially regulated genes compared to CTR-NT. This lack of effect of TSA could be considered in the light of three facts: i) the epigenetic reprogramming mediated by oocyte reprogramming factors is much more efficient than TSA [16], ii) following epigenetic alteration by TSA, somatic cell nuclei are still refractory to the epigenetic reprogramming [38], and iii) SCNT-specific transcriptional errors are of a non-random nature and are responsive to neither oocyte- nor TSA-mediated reprogramming [39].

Whether specific loci in the genome are preferentially affected during SCNT (reprogramming error hotspots) or the SCNT errors have a stochastic nature is still an elusive controversial question [39, 40]. Consistent deregulation of a defined list of genes would support the hotspot scenario whereas a stochastic model of reprogramming would be suggested when an unpredictable list of genes would emerge from different SCNT runs [40]. Our microarray data revealed consistent and predictable profiles of gene expression between the two SCNT groups irrespective of TSA treatment. Accordingly, almost all downregulated genes and a great portion of upregulated genes in either NT group were similarly expressed in the other NT group. Moreover, as much as 15 % of the genes consistently downregulated in both NT groups were located in chromosome X. This predictability of individual gene expression on a global background of multiple gene expression changes argues for a predominantly non-random, rather than stochastic, nature of reprogramming errors in bovine SCNT embryos which is in agreement with the study of Inoue et al. [39] on mouse SCNT. Similarly, Maruotti et al. [41] observed a clear bias in the distribution of differentially expressed genes between IVF and NT-derived epiblast stem cells in mice with 30 % of DEGs being localized on chromosome 11. However, Somers et al. [40] observed different embryo to embryo expression profiles in bovine SCNT. Although we have used several pools of embryos for each contrast, a defined pattern of differences indicates, at least for our fibroblast line, non-random reprogramming errors. While this manuscript was being prepared for publication, a study appeared by Matoba et al. [42] that identified genomic domains resistant to ZGA in mouse SCNT by using comparative transcriptome analysis. Because these so-called reprogramming resistant regions (RRRs) were enriched for H3K9me3 in somatic nuclei, and since mRNA injection of Kdm4d markedly improved SCNT blastocyst rate from 26.0 to 86.6 %, they introduced H3K9me3 as the critical epigenetic barrier in SCNT-mediated reprogramming in mouse. Importantly, combined treatment with Kdm4d mRNA and TSA improved cloning efficiency compared to alone treatment with TSA, but not Kdm4d. They suggested that TSA and Kdm4d have no synergic effect because their effects are modulated through a similar pathway. This study of Matoba et al. [42] in mouse may support our hypothesis on non-random nature of oocyte-mediated reprogramming errors in bovine. Therefore, a clear understanding of RRRs in different differentiated/differentiating cells and whether RRRs are of a common or specific-specific nature in mammals will provide a promising approach for improving the efficiency of SCNT and induced pluripotent stem cell (iPS) technologies.

The great number of X-linked genes consistently deregulated in either group may highlight the importance of reprogramming difficulties on this chromosome. Among the deregulated genes, IGF2R was upregulated and NAP1L5 and XIST were downregulated in both NT groups compared to IVF. Although both X chromosomes are active in the female zygote, one X chromosome endures a dosage compensation mechanism of transcriptional silencing that proceeds through a complex sequence of events between hatching and implantation in bovine embryos [43, 44]. Reconstituted SCNT embryos, however, receive one active and one inactive X chromosome from the donor cell and hence compromising the natural pattern of imprinting reprogramming [44]. Interestingly, while Eggan et al. [45] reported a normal pattern of X chromosome inactivation (XCI), Xue et al. [46] reported aberrant expression of nine of ten X-linked genes and hypomethylation of XIST in organs of deceased cloned calves. Similarly, siRNA-mediated knockdown of XIST resulted in a drastic (>10-fold) increase in the birth rate of male clones [47]. In a subsequent study the same group showed that although XIST siRNA injection largely suppressed over-expression of XIST in female SCNT embryos, it could not improve survival [48]. It is worth noting that after TSA treatment of female bovine embryos, Olivera et al. [44] observed an 8-fold decrease in the percentage of blastocysts expressing detectable XIST, whereas the expression of G6PD remained unaltered, suggesting specific susceptibility of XIST to *in vitro* culture conditions.

The establishment of ESCs analogous to those derived from human and mice embryos has remained an elusive goal in ungulates [49]. A clear knowledge of the transcriptional regulation of pluripotency genes is fundamental to the understanding of the species-specific characteristics involved and might reveal clues to achieve stable ESCs in ungulates. Analysis of the mouse embryo has revealed that several of the genes that are implicated in the divergence of the ICM are identifiable within the context of five canonical pathways, namely TGF-β/activin-nodal, FGFR, WNT, BMP and Notch (http://www.cellsignal.com/). These gene regulatory networks provide the molecular foundation for the stabilization or destruction of the core pluripotency triad (OCT4, SOX2, and NANOG). Examination of genes involved in different canonical pathways revealed that a typical bovine SCNT blastocyst may possess functional WNT and to some extent FGF signaling pathways, but TGF-β and BMP pathways are not functional. Notably, while TSA had no apparent effect on WNT and FGF signal transduction pathways, it altered the expression of SMADs. Conversely, Denicol et al. [50] demonstrated the expression of sixteen WNT genes and other genes involved in WNT signaling in Day 6 morulae, indicating a functional WNT signaling system. By applying WNT agonists and antagonists, they provided evidence suggesting that the WNT signaling pathway is conversely associated with preimplantation embryonic development in bovine. Despite similarities in the WNT and FGF pathways, CTR-NT and TSA-NT -derived blastocysts differed in the expression patterns of SMADs. While the contributions of SMADs to pluripotency and the downstream events that they may regulate are poorly understood, the observed bias in the expression profile of SMADs may be associated with the differences observed in the level of downregulation of NANOG in TSA-NT- and CTR-NT-derived blastocysts (4- and 1.7-fold, respectively). The homeobox NANOG is among the highly conserved transcription factors implicated in ESC identity, self-renewal and maintenance, in cooperation with OCT4 and SOX2. Therefore, the observed reduction in the expression of NANOG should have important implications in the development of cloned embryos. In contrast, Iager et al. [19] reported increased expression of NANOG in SCNT bovine embryos treated with TSA. The transcription factor C-MYC is central to a module of highly connected binding sites that are related to different epigenetic modifications of the core pluripotency-associated network [51]. C-MYC is expressed in ESC and cancer cells and it was also upregulated in both SCNT groups in this study. However, C-MYC functions seem to be distinct from the pluripotency triad’s functions and it may be more involved in the regulation of protein metabolism [51]. Double inhibition of MAPK2 and GSK3 in bovine blastocysts increased NANOG and SOX2, but not C-MYC [52]. Taken together, the simultaneous expression of both stimulatory and inhibitory pathways may put the pluripotency of SCNT blastocysts in a precarious contradictory state compared to IVF counterparts.

Abnormal placental development has been deemed responsible for the majority of SCNT problems [53–56]. It is believed that dysfunctional placentation in clones is the result of aberrant expression of several key transcription factors that are involved in proliferation (MASH2, ERG, CDX2, TBPG), differentiation (CDX2, ERG, LOC618696, PLET1, POU5F1, HAND1) and function (ERG, ERG, IFN-tau, TKDP1, PAG-9) of trophoblasts [55, 56]. Importantly, expression of some of these transcripts is increasingly enhanced as the bovine trophoblast elongates [55]. In our results, TKDP1, LOC618696 and TBPG were downregulated over 3-fold in SCNT blastocysts of both groups, IFN-tau was downregulated in both groups, and HAND1 was downregulated in the TSA-NT group. TKDP1 is responsible for trophoblastic Kunitz domain proteins which are is assumed to protect the developing conceptus against potentially damaging proteinases released by maternal cells [57]. Therefore, one may argue that downregulation of these genes may be involved in the early pregnancy losses.

It has been demonstrated that assisted epigenetic modification improves reprogramming efficiency through improvement of nuclear organization and induction of synchrony between the blastomeres for the expression of nascent mRNA [26, 27]. We therefore attempted to follow the dynamics of nuclear remodeling and nascent mRNA expression in association with the epigenetic alterations of the reconstituted oocytes and blastocysts. The most notable finding was that TSA treatment resulted in a very sharp drop in the level of 5mC (by 50 %) immediately after nuclear transfer coinciding with a parallel increase in the level of 5hmC (by 4-fold). Interestingly, the 5mC signal at 24 hpr was comparable to the level observed in early reconstituted oocytes. However, the 5hmC signal remained significantly higher in TSA-NT vs. CTR-NT embryos. Similarly, Antony et al. [58] showed that although induction of jmjd2b in mice ESC substantially decreased total levels of H3K9me3 by 63 %, the level of H3K9me3 was returned to normal within minutes following fusion with an enucleated oocyte. Notably, this transient change in H3K9me3 resulted in a remarkable increase in *in vitro*, but not in vivo, development which is in agreement with our results. The second finding was that the global levels of DNA-methylation and H3K9 acetylation in TSA-NT blastocysts were modified to levels similar to IVF blastocysts and this is in agreement with all the other studies [16–19, 31]. It remains enigmatic how TSA could affect blastocyst formation but not post-implantation health.

These dramatic changes in initial (5mC, and 5hmC) and final (DNA-methylation and H3K9 acetylation) levels of these crucial epigenetic markers did not recapitulate the changes in chromatin remodeling and epigenetic genes expression. Notably, the time-window of nascent OCT4 and nascent mRNA expression was not altered by TSA treatment. In agreement, using GFP-expressing fibroblasts fused to oocytes reconstructed either before (metaphase) or after (telophase) activation, Bordignon et al. [59] could not detect GFP signals until the 8- to 16-cell stage regardless of the cytoplast used, suggesting equivalent functional remodeling of chromatin activity in embryos reconstructed with both types of cytoplasts. In contrast, Bui et al. [26] and VanThuan et al. [27] demonstrated a more effective formation of DNA replication complexes in TSA- and Scriptaid-treated mouse embryos which was evidenced by enhanced levels of newly synthesized RNA and significant reduction in asymmetric expression of nascent RNA. Based on our previous study on TSA treatment of early reconstituted embryos [18], this controversy may not be related to TSA treatment of nuclei donor cells or early reconstituted oocytes but instead may be related to species-specific differences in the timing of zygotic genome activation (ZGA) (2-cell in mice and 8–16-cell in bovine [1]). These differences provide an unwanted variability in the timing of assisted epigenetic modification in relation to the timing of maximum epigenetic alteration and ZGA.

## Conclusions

This is the first comprehensive study on the effects of assisted epigenetic modifications on the transcriptome of bovine SCNT blastocysts studied with a microarray chip which covers the bovine preattachment transcriptome and hence allows the exploration of gene expression in bovine blastocysts obtained in different conditions. The results obtained demonstrated substantial reprogramming of the donor cell nuclei by the blastocyst stage with no overt effect of TSA on the number and identity of differentially regulated genes. Considering the limited number of genes deregulated in SCNT embryos, one may suggest that a simple defect in the expression of a few genes may translate into defective expression of a great number of genes in a ripple effect. This may also suggest a non-random nature of SCNT-specific errors that are not TSA-responsive. This also suggests that the miracle of cloning lies in the great capacity of the ooplasm to reprogram almost any type of differentiated cells; but the problems of cloning are due to unstable genes that escape/resist reprogramming and are not responsive to TSA. Therefore, future studies should focus on these potentially important deregulated genes involved in extra-embryonic tissue modelling to find strategies to correct their expression before the embryos are transferred to recipients.

## Methods

Unless otherwise specified, all chemicals and media were obtained from Sigma-Aldrich (St. Louis, MO, USA) and Gibco (Life Technologies, Rockville, MD, USA), respectively. All animal care and surgical procedures were undertaken in strict accordance with the approval of the Institutional Review Board and Institutional Ethical Committee of Royan Institute (No#94321).

### Experimental design

The objective of this study was to investigate the effects of TSA treatment of donor cells on the transcriptional profile of cloned embryos compared to control SCNT and IVF embryos (hereafter TSA-NT vs. CTR-NT vs. IVF, respectively) and to identify specific genes or categories of genes that could account for specific mechanisms or events that occur in somatic cells, reconstituted oocytes and developing embryos. To meet these objectives, an established fetal fibroblast cell line obtained from an approximately 60-day female fetus, was treated with a previously established concentration and duration of TSA (1.0 μM for 24 h, [60]). At the end of each treatment, treated and untreated (control) cells were used for either SCNT or flow-cytometery assisted analyses of DNA-methylation and histone acetylation. Yield and quality of embryo development was recorded until day 7 when grade 1 & 2 blastocysts were used for microarray analysis, embryo transfer to recipients and differential staining. Further, some of the SCNT and IVF embryos were collected at four intervals post SCNT/IVF (0, 4, 8, 12, 24 h) for immunostaining assessment of epigenetic marks and chromatin remodeling using immunostaining against 5-methylcytosine (5mC), 5-hydroxymethylcytosine (5hmC) and β-tubulin, respectively. Other embryos were also collected for the assessment of nascent mRNA expression using immunostaining against BrUTP. Also, fibroblasts were stably transfected with a construct of EGFP-OCT4 [16] for the detection of the time-window of the expression of genes for pluripotency in TSA-NT and CTR-NT embryos. Finally, qRT-PCR was used to determine the effects of TSA on the expression profile of a subset of genes in fibroblast cells and resulting embryos.

### Embryo production by IVF

For *in vitro* maturation (IVM), cumulus-oocyte complexes (COCs) were obtained by aspirating antral follicles (2–8 mm in diameter) of ovaries obtained from an abattoir. Groups of ten COCs with homogenous cytoplasm and more than three layers of compact cumulus cells were placed in 50-μl drops of maturation medium (MM) under mineral oil and matured for 24 h at 39 °C under an atmosphere of 6 % CO_2_ in air with maximum humidity. The MM was comprised of tissue culture medium 199 (TCM199) supplemented with 2.5 mM Na-pyruvate, 100 IU/ml penicillin, 100 μg/ml streptomycin, 1 mg/ml estradiol-17β, 10 μg/ml FSH, 10 μg/ml LH, 100 ng/ml EGF, 100 ng/ml FGF, 0.1 mM cysteamine, and 10 % fetal calf serum (FCS) at 39 °C, 6 % CO_2_. [61].

For fertilization, commercially available frozen semen straws were thawed (30 s in air and then 1 min in 37 °C water) and the semen was centrifuged (70 g for 5 min) to remove the cryoprotectants. The semen pellet was layered upon a discontinuous PureSperm® gradient (2 ml of 45 % over 2 ml of 90 % prepared in hepes TCM199 (HTCM199)) and motile sperm were collected after centrifugation (1200 g, 20 min) at room temperature (RT). Prepared samples were washed twice in hepes-buffered Tyrode’s albumin lactate pyruvate medium and 2 × 10^5^ sperm were co-incubated with ten COCs in 200 μl fertilization medium for 18–20 h at 39 °C, 6 % CO_2_ and humidified atmosphere. The presumptive zygotes were then denuded of their cumulus cells and washed twice with embryo culture medium. Since the SCNT procedure used in this study was a zona-free procedure, in order to alleviate potential bias in the array comparison of SCNT and IVF blastocysts, the zona pellucida was also removed from IVF zygotes before culture for *in vitro* embryo development [16].

For embryo culture, a continuous modified synthetic oviduct fluid (mSOF) containing 100 ng/ml EGF, 0.5 mM glucose, and 2 μl/ml ITS (insulin, transferrin and selenium) was used. To preclude aggregation of zona-free embryos, 50-μl droplets of mSOF were prepared under embryo-tested mineral oil in 3 cm Grainer® dishes. Next, ten small wells were made by gently pressing a sterile steel rod with a round tip (500 μm diameter) to the bottom of the culture dish and the presumptive zygotes were put in separate wells. The embryo culture dishes were incubated at 39.0 °C with 6 % CO_2_, 5 % O_2_, 90 % N_2_ and maximum humidity for 7 days. The numbers of embryos that cleaved and developed to the blastocyst stage were recorded at day 3 and 7 post-IVF, respectively [16].

### Embryo production by SCNT

For somatic cell culture, a skin biopsy from an approximately 60-day old female fetus was used as the source of bovine fetal fibroblasts as described previously [60]. After thorough washing with phosphate buffer saline (PBS) containing antibiotics and antimycotics, the biopsy was cut into small pieces (2–3 mm^2^), and the explants were cultured in Dulbecco’s modified Eagle’s medium F-12 (DMEM/F-12) containing 10 % FCS and 1 % penicillin-streptomycin at 37 °C in a humidified atmosphere of 5 % CO_2_ until confluence. To confirm the fibroblast phenotype, cells at passage two were immunostained against anti-vimentin (for fibroblasts) and anti-pancytokeratin (for epithelial cells). For TSA treatment, 4 × 10^5^ fibroblasts at passage three were added to 3-cm culture dishes containing DMEM/F-12 plus 10 % FCS supplemented with either 1.0 (treatment) or 0.0 (control) μM of TSA and cultured for 24 h before being trypsinized for SCNT or flowcytometry assessment of epigenetic marks as described elsewhere [60].

For oocyte reconstitution, denuded oocytes were released from their zona pellucida by brief incubation (up to 45 s) in 5 mg/ml pronase dissolved in HTCM199 containing 10 % FCS. Enucleation of the oocytes was performed in PBS free of Ca^2+^ and Mg^2+^ and supplemented with 20 % FCS, 2.5 mM Na-pyruvate, 1 % bovine serum albumin (BSA), 1 % polyvinyl alcohol (PVA) and 1.5 mM glucose. Enucleation was carried out at 100X magnification on a pre-warmed microscope stage (Olympus, IX71) under constant UV-light exposure with the help of blunt perpendicular-break enucleation pipettes (15–20 μm inner diameter). Fibroblast cells were trypsinized immediately before NT, and a low density of cells was prepared in a drop of HTCM199 + 0.5 % FCS containing 10 μg/ml phytohemagglutinin. Five to ten enucleated oocytes were added to the droplet, and each oocyte was gently pushed over a single cell. The oocyte-donor cell couplets were placed between two electrodes (0.5 mm apart), overlaid with a hypo-osmotic fusion medium (0.2 M mannitol, 100 μM MgSO_4_, 50 μM CaCl_2_, 500 μM hepes, and 0.05 % BSA), aligned first manually and then by application of AC current (7 V/cm, 1,000 KHz, for 10 s). Fusion was induced by two successive DC current pulses (1.75 KV/cm, 30 μsec with 100 μsec interval) [16]. Fused couplets were activated within 30 min of electro fusion by incubation in 5 μM calcium-ionophore for 5 min, followed by 4 h exposure to 2 mM 6-dimethylaminopurine dissolved in TCM199 containing 10 % FCS, 0.2 mg/ml PVA, 3 mg/ml BSA [62]. Activated embryos were cultured and evaluated as described for the IVF embryos.

### Quality assessment of blastocysts

For quality assessment of embryo development, blastocysts were graded according to the guidelines of the International Embryo Transfer Society (IETS). Since it has not been established whether these grading criteria are at all meaningful for zona-free embryos or SCNT embryos, we also relied on our own experience [16, 18, 60]. For further assessment of embryo quality, differential staining to detect total (TCN), inner (ICM) and trophectoderm (TE) cell numbers was carried out as described previously [61]. In brief, blastocysts were incubated in 500 μL of 1 % Triton X-100 and 100 μg/mL propidium iodide for up to 30 s depending on the size of the embryos, and then immediately transferred to 500 μl of a solution of 100 % ethanol plus 25 μg/mL H33342 and stored at 4 °C overnight. Fixed and stained embryos were subsequently mounted on glass microscope slides in one drop of glycerol, gently flattened with a coverslip, and visualized for cell counting on a fluorescence microscope (Olympus, BX51) using the 460 nm excitation filter for blue and the 560 nm filter for red. TE cells were visualized as pink and ICM as blue. The TCN of each embryo was calculated by adding the number of ICM and TE.

### RNA isolation, amplification, and microarray hybridization

The whole process of microarray analysis was as described previously [63]. Total RNA from each replicate was extracted and purified using the PicoPure RNA Isolation Kit. After DNase digestion (Qiagen), the quality and concentration of the extracted RNA was determined with a bioanalyzer (Agilent). All extracted samples were of good quality with RNA integrity numbers ≥7.0.

For microarray purposes, purified RNA was *in vitro* transcribed by T7 RNA amplification using the RiboAmp HS^Plus^ RNA Amplification Kit (Life Science) and labeled with Cy3 and Cy5 using the ULS Fluorescent Labeling Kit (Kreatech). Antisense RNA (825 ng per replicate) was then hybridized on the Agilent-manufactured EmbryoGENE® slides [30] in a two-color dye-swap design. The microarray chip used, EmbryoGENE®, covers most of the bovine pre-attachment transcriptome and hence allows the analysis of gene expression in bovine blastocysts.

After 17 h of hybridization at 65 °C, microarray slides were washed for 1 min in gene expression wash buffer 1 (RT), 3 min in gene expression wash buffer 2 (42 °C), 10 s in 100 % acetonitrile (RT) and 30 s in Stabilization and Drying Solution (Agilent). Slides were scanned with a Power Scanner (Tecan) and features extraction was done with Array-pro6.3 (Media Cybernetics). Intensity files were analyzed with FlexArray 1.6.1 (Michal Blazejczyk, Mathieu Miron, Robert Nadon (2007), FlexArray: statistical data analysis software for gene expression microarrays. Genome Quebec, Montreal, Canada, URL: http://genomequebec.mcgill.ca/FlexArray). Specifically, raw data were corrected by background subtraction, and then normalized within and between each array (Loess and quantile, respectively). Statistical comparison between treatments (CTR-NT vs. IVF and TSA-NT vs. IVF) was done with the Limma algorithm. In pairwise comparisons among transcripts with a *p*-value < 0.01, a false discovery rate (FDR) of 20 % and a fold change >2.0 were considered differentially expressed. The data discussed in this publication have been deposited in NCBI’s Gene Expression Omnibus and are accessible through GEO Series accession number GSE57247 under URL: http://www.ncbi.nlm.nih.gov/geo/query/acc.cgi?acc=GSE57247

#### Validation of microarray results by qRT-PCR

Microarray results were validated by quantitative real-time PCR (qRT-PCR) as described previously [16]. The list of selected primers with their characteristics is shown in Additional file 9: Table S8. In brief, total RNA was extracted from independent samples (groups of ten blastocysts in three replicates for each experimental group) using RNeasy Micro Kit (Qiagen, Hilden, Germany) according to the manufacturer’s guidelines. The concentration of the extracted RNA was determined by measuring the absorbance at 260 nm using a spectrophotometer and total extracted RNA (around 0.5 μg) was used for first strand cDNA synthesis with the RevertAid® First Strand cDNA Synthesis Kit (Fermentas, Germany). Each cDNA synthesis reaction contained 1 μl random hexamer primer, 1 μl RNase inhibitor, 4 μl 5X reaction buffer, 2 μl dNTP, and 1 μl M-MuLV reverse transcriptase and was adjusted to a total volume of 20 μl using DEPC-treated water. The synthesis of cDNA was performed at 42 °C for 1 h. The qRT-PCR was carried out using the Rotor Gene 6000 (Corbett®, Australia). Each reaction mix contained 2 μl cDNA, 10 μl SYBR Premix Ex Taq II (TaKaRa®, Japan) and 1 μl of 5 pM/ml forward and reverse primers and adjusted to a total volume of 20 μl using dH_2_O. The primers were designed using Beacon Designer, checked by NCBI blast, and finally by gel electrophoresis. The efficiencies of the designed primer pairs were checked by amplification of a serial dilution of template cDNA (50, 10, 2, 0.4, and 0.08 ng) and were between 90 % and 100 %. The obtained CT of each target gene was normalized to the CT of a housekeeping gene (ACTB) and represented with reference to IVF as 2^-ΔΔCT^.

#### Functional analysis of differential gene expression profiles

The Ingenuity Pathways Analysis (IPA; Ingenuity Systems, www.ingenuity.com) software was used to group overrepresented functions of differentially expressed genes into clusters. Moreover, IPA was queried to compile canonical pathways as well as gene regulatory networks (GRNs) that were differentially expressed between treatments. We used IPA to build schematic representations of important pathways deregulated in SCNT blastocysts [63].

#### Network generation

A data set containing the gene identifiers and the corresponding expression values was uploaded into the application. Each identifier was mapped to its corresponding object in Ingenuity’s Knowledge Base. A fold-change cut-off of 1.5 with a *P* value <0.05 was set to identify molecules whose expression was significantly differentially regulated. These molecules, called network-eligible molecules, were overlaid onto a global molecular network developed from information contained in Ingenuity’s Knowledge Base. Networks of network-eligible molecules were then algorithmically generated based on their connectivity. Molecules are represented as nodes, and the biological relationship between two nodes is represented as an edge (line). All edges are supported by at least one reference from the literature, from a textbook, or from canonical information stored in the Ingenuity Pathways Knowledge Base. Human, mouse, and rat orthologs of a gene are stored as separate objects in the Ingenuity Pathways Knowledge Base, but are represented as a single node in the network. Nodes are displayed using various shapes that represent the functional class of the gene product [63].

#### Canonical pathway analysis

Canonical pathway analysis identified the pathways from the IPA library of canonical pathways that were most significant to the data set. The significance of the association between the data set and the canonical pathway was measured in two ways: 1) a ratio of the number of molecules from the data set that map to the pathway divided by the total number of molecules that map to the canonical pathway was displayed; 2) Fisher exact test was used to calculate a *P* value to determine the probability that the association between the genes in the dataset and the canonical pathway would occur by chance alone. Green and red symbols represented genes respectively down and upregulated in treated embryos compared to controls. Gray symbols represented genes with significant expression in blastocysts but with no difference between conditions, whereas white symbols represented genes not present on microarray or with below-background intensity [63].

### Immunofluorescence

#### DNA-methylation and histone H3K9 acetylation in fibroblasts

As described previously [60], quantitative assessment of DNA-methylation and histone H3K9 acetylation was conducted by incubating fibroblasts with 1:400 and 1:200 dilutions of mouse anti-5-methylcytosine and anti-H3K9 monoclonal antibodies, respectively. Fluorescein isothiocyanate-conjugated goat anti-mouse immunoglobin was used as the secondary antibody at a 1:50 dilution. A corresponding control for each experiment was included. Cells were filtered through a 40-μm nylon mesh in order to exclude aggregated cells. Ten thousand cells were collected with the fluorescence activated cell sorting (FACS)-Caliber and were analyzed using CELL QUEST® 3.1 software (Becton Dickinson). Three replicates were conducted for each treatment with appropriate controls to eliminate the possible effects of auto fluorescence and non-specific binding by the secondary antibody.

#### DNA-methylation and histone H3K9 acetylation of blastocysts

As described previously [16], NT and IVF blastocysts were washed in PBS containing 1 mg/ml PVA and then fixed in 4 % paraformaldehyde (PF) in PBS for 30 min. Permeabilization was carried out in 0.5 % Triton X-100 in PBS for 15 min. For DNA-methylation, embryos were first pre-treated with 4 N HCl for 60 min at RT, and then washed with PBS. To preclude non-specific binding of the primary antibody, embryos were treated with 3 % BSA in PBS for 60 min at RT. These embryos were then incubated with the primary antibody: either mouse monoclonal anti-5-methylcytosine (for DNA-methylation) (Eurogentec®, BI-MECY-0100) or mouse monoclonal anti-H3K9 (for histone-acetylation), for 1 h at 37 °C, followed by three washes in PBS containing PVA. Embryos were then incubated with the secondary antibody (goat anti-mouse IgG-TRITC conjugate) for 60 min at 37 °C. Subsequently, embryos were re-fixed overnight in 4 % PF and treated with 0.1 mg/ml RNase-A for 1 h before being washed in PBS. The fixed and stained embryos were mounted and imaged as described for EGFP fluorescence using 557 nm excitation and 576 nm emission filters. The median pixel intensity of 10–15 nuclei was detected in each NT and IVF blastocyst. Briefly, five different regions of each image that contained 10–15 nuclei were randomly selected and the average pixel intensity of fluorescence emission in the nuclei located in these regions was detected using Image J. software (National Institute of Mental Health, Bethesda, Maryland, USA). The software was zeroed against background before use. Appropriate controls were also included to eliminate the possible effects of auto fluorescence of the first and second antibodies.

#### Chromatin remodeling dynamics

Stepwise assessment of chromatin remodeling was carried out as described previously [64]. In brief, reconstituted oocytes at 0.5–12 h post-reconstitution (hpr) were fixed in 4 % PF for 15 min. Microtubules were immunostained with anti-β-tubulin monoclonal primary antibody (1:100) and FITC labeled anti-mouse-IgG secondary antibody (1:100). The chromosomes were counterstained with H333242 (2 mg/ml) and then samples were washed and mounted on glass slides in glycerol droplets to be observed with an epifluorescence microscope (Olympus, BX51) at 400X magnification. A digital image of each sample was taken with a highly sensitive camera (Olympus DP-72) operated on DP2-BSW Software.

#### 5mC and 5hmC dynamics

The disappearance of 5mC and appearance of 5hmC were assessed as described previously [65]. Briefly, reconstituted oocytes and IVF embryos at 0, 4, 8, 12, and 24 h post SCNT/IVF were fixed in 4 % PF for 10 min at RT, washed several times in PBS-Tween (PBS-T: 0.1 %) and then permeabilized in 1 % Triton X-100/PBS-T for 1 h. Tissues were washed again in TBS-T then antigens were blocked by incubating in 5 % non-fat dry milk/TBS-T for 1 h at RT. First antibodies were added to fresh blocking solution at 1:1000 and 1:2000 dilutions for anti-5mC and anti-5hmC, respectively. Samples were incubated overnight at 4 °C in antibody solution, and then washed in PBS-T several times. Goat anti-mouse secondary antibody was diluted at 1:2000 in freshly made blocking solution. Samples were incubated for 45 min followed by four washes of 15 min in PBS-T and incubated in DAPI 1:1000 for nuclear staining. Samples were washed and mounted on glass slides in glycerol droplets to be observed with an epifluorescence microscope (Olympus BX51) as described for chromatin remodeling.

#### Nascent mRNA expression

Incorporation of BrUTP into embryos was performed as described by Bui et al. [26]. In brief, SCNT embryos at different stages of *in vitro* development (oocyte, 2-, 4-, 8–16 cells, compact morula and blastocyst) were washed trice with electrical permeabilization buffer (PB: 0.25 M D-glucose, 100 μM CaCl_2_.2H_2_O, 100 μM MgSO_4_, and 0.1 % PVP) and then incubated in transcription buffer (PB + 10 mM BrUTP) for 1 min. Embryos were placed between two electrodes (0.5 mm apart) of an electro fusion chamber filled with 20-μL droplets of transcription buffer and BrUTP internalization was induced by two successive DC current pulses (250 V/cm, 80 μsec with 1 min interval). Embryos were transferred to mSOF medium two minutes after permeabilization and cultured for 1 h. The embryos were then washed twice in PBS-PVA, fixed for 40 min in PBS-PVA, washed twice in PBS-PVA and stored overnight at 4 °C in PBS supplemented with 3 % BSA and 0.1 % Triton X-100. The embryos were then incubated for 2 h with mouse monoclonal anti-BrdU (6 μg/mL), washed twice in PBS-BSA before being incubated for 1 h in 1:200 dilution of Tritc-labeled goat anti-mouse IgG as second antibody. Embryos were then washed out trice in PBS-BSA, 10 min each, and observed with high power inverted and then epifluorescence microscopes as described above. Blastocysts and oocytes were used as positive and negative controls, respectively.

#### Nascent EGFP-OCT4 expression

Fibroblasts were transfected with a previously produced EGFP-POU5F1 plasmid containing a neomycin resistance gene via lipofection method (LipofectAMINE 2000^TM^). The EGFP-POU5F1 vector map and the transfection protocol are available in a previous article [18]. Stably transfected colonies, selected after antibiotic screening and PCR, were expanded before being used for SCNT. The time window and the extent of EGFP-OCT4 expression in the developed embryos at the 4- and 8–16-cell, morulae, and blastocyst stages were assessed by inverted and then epifluorescent microscopy.

### Identification of the effects of TSA on fibroblast gene expression

To identify the effects of TSA on fibroblast gene expression, microarray data were queried to select a subset of non-DEG transcripts in both NT groups that were oppositely expressed between CTR-NT and TSA-NT blastocysts. The primer list of selected genes is shown in Additional file 9: Table S8. Then, based on a design described by Whitworth et al. [28], qRT-PCR was used to compare the expression profiles of this subset of genes in TSA-treated and untreated fibroblasts, and CTR-NT, TSA-NT, and IVF blastocysts.

## Additional files

Additional file 1: Table S1. Pre- and post- implantation development. Effect of TSA treatment on in vitro and in vivo development of cloned embryos compared to fertilized counterparts. (DOCX 18 kb) Additional file 2: Table S2. CTR-NT Differentially regulated genes. Breakdown of genes differentially up and downregulated with ≥1.5 fold change in CTR-NT blastocyst compared to in vitro fertilized embryos. Red and green colors represent up and downregulation, respectively. (XLS 261 kb) Additional file 3: Table S3. TSA-NT differentially regulated genes. Breakdown of genes differentially up and downregulated with ≥1.5 fold change in TSA-NT compared to in vitro fertilized embryos. Red and green colors represent up and downregulation, respectively. (XLS 314 kb) Additional file 4: Table S4. Unique gene expression in CTR-NT and TSA-NT. A breakdown of genes predominantly or commonly expressed in CTR-NT- and TSA-NT-derived blastocysts. (XLS 416 kb) Additional file 5: Figure S1. Nascent RNA expression in CTR-NT and TSA-NT blastocysts. A-E’) Investigation of nascent RNA expression at different stages of cloned embryo development. Oocytes and blastocysts are considered as negative and positive controls based on RNA expression, respectively. No sign of mRNA expression was observed until the 8 (C&C’) -16 (D&D’) cell stage. Scale bar represents 100 μm. (JPEG 594 kb) Additional file 6: Table S5. GO terms emerged from CTR-NT blastocyst. Gene ontology terms emerged from the differentially regulated genes of CTR-NT blastocysts compared to IVF (FDR 1.5, P ≤ 0.05, FC ≥1.5). (XLS 39 kb) Additional file 7: Table S6. GO terms emerged from TSA-NT blastocyst. Gene ontology terms emerged from the differentially regulated genes of TSA-NT blastocysts compared to IVF (FDR 1.5, P ≤ 0.05, FC ≥1.5). (XLS 42 kb) Additional file 8: Table S7. Real Time PCR gene expression analysis. Validation of microarray results by qRT-PCR quantification of the mRNAs in blastocysts developed in CTR-NT and TSA-NT groups compared to IVF. The analysis was done in five replicates of pools of ten blastocysts. ACTB was considered as housekeeping gene. (DOCX 17 kb) Additional file 9: Table S8. qRT-PCR primers. Sequences (5′–3′) of reverse transcription qRT-PCR-specific primers for candidate genes expressed in bovine embryos. (DOCX 19 kb)

## Abbreviations

SCNT: Somatic cell nuclear transfer
TSA: Trichostatin A
IPA: Ingenuity Pathways Analysis
PCA: Principal component analysis
CTR: Control
NT: Nuclear transfer
IVF: In vitro fertilization
5mC: 5 methyl cytosine
5hmC: 5 hydroxymethyl cytosine
DEG: Differentially expressed genes
mSOF: Modified synthetic oviduct fluid
qRT-PCR: Quantitative real-time PCR
EGFP: Enhanced green fluorescent protein
FCS: Fetal calf serum
BSA: Bovine serum albumin
PVA: Polyvinyl alcohol
PBS: Phosphate buffer saline
RT: Room temperature
FACS: Fluorescence activated cell sorting
RRRs: Reprogramming resistant regions
