# Supplementary material for: Epigenetic modification with trichostatin A does not correct specific errors of somatic cell nuclear transfer at the transcriptomic level; highlighting the non-random nature of oocyte-mediated reprogramming errors
Source: BMC Genomics. 2016 Jan 4;17:16. doi: 10.1186/s12864-015-2264-z (PMC4698792; doi:10.1186/s12864-015-2264-z)
Supplement: Additional file 1: Table S1. — Pre- and post- implantation development. Effect of TSA treatment on in vitro and in vivo development of cloned embryos compared to fertilized counterparts. (DOCX 18 kb) [file 12864_2015_2264_MOESM1_ESM.docx]

| **Additional file 1: Table S1. Effect of TSA treatment on in vitro and in vivo development of cloned embryos compared to fertilized counterparts.** | | | | | |
| --- | --- | --- | --- | --- | --- |
|  |  |  | **Treatments** | | |
|  | **Comparisons** |  | **CTR-NT (%±SEM)** | **TSA-NT (%±SEM)** | **IVF (%±SEM)** |
| **(A)**  **In vitro development** | **No. of embryos** |  | 3610 | 2689 | 1254 |
|  | **Cleavage** |  | 3332 (92.3±7.8) | 2265 (85.7±9.5) | 986 (78.6±4.6) |
|  | **Blastocyst** |  | 956 (28.7±5.5) a | 909 (39.8±4.1) b | 338 (34.3±3.9) ab |
|  | **Grade 1&2 blastocysts** |  | 332 (34.7±6.7) a | 436 (48.0±6.0) b | 141 (41.7±4.2) ab |
|  |  |  |  |  |  |
|  |  |  |  |  |  |
| **(B)**  **Differential staining** | **No. of blastocysts** |  | 12 | 15 | 21 |
|  | **No. of ICM** |  | 25.0±6.9 a | 36.1±9.7 b | 31a |
|  | **No. of TCN** |  | 103.4±11.1 | 123.3±14.3 | 109.6±8.9 |
|  | **% of ICM/TCN** |  | 0.22±3.3 a | 0.35±4.5 b | 28.6±7.5 ab |
|  |  |  |  |  |  |
| **(C)**  **In vivo development** | **No. of embryos transferred/recipients** |  | 205/67 | 159/56 | 44/28 |
|  | **D35-40 gestation** |  | 31 (46.3) | 21 (39) | 10 (35.7) |
|  | **D60-65 gestation** |  | 12 (38.7)a | 6 (30)a | 9 (90.0)b |
|  | **D90-120 gestation** |  | 9 (75.0)ab | 4 (65)a | 9 (90.0)b |
|  | **Terminated due to complications** |  | 3 (33.3)a | 2 (50)a | 0b |
|  | **Still birth** |  | 4 (66.7)a | 1 (50)a | 0b |
|  | **Survived** |  | 2 (66.7)a | 1 (50)a | 9 (100.0)b |
| In each row, values with different letters differ significantly (p<0.05). | | | | | |
