# Supplementary figures and images for: Epigenetic modification with trichostatin A does not correct specific errors of somatic cell nuclear transfer at the transcriptomic level; highlighting the non-random nature of oocyte-mediated reprogramming errors
Source: BMC Genomics. 2016 Jan 4;17:16. doi: 10.1186/s12864-015-2264-z (PMC4698792; doi:10.1186/s12864-015-2264-z)

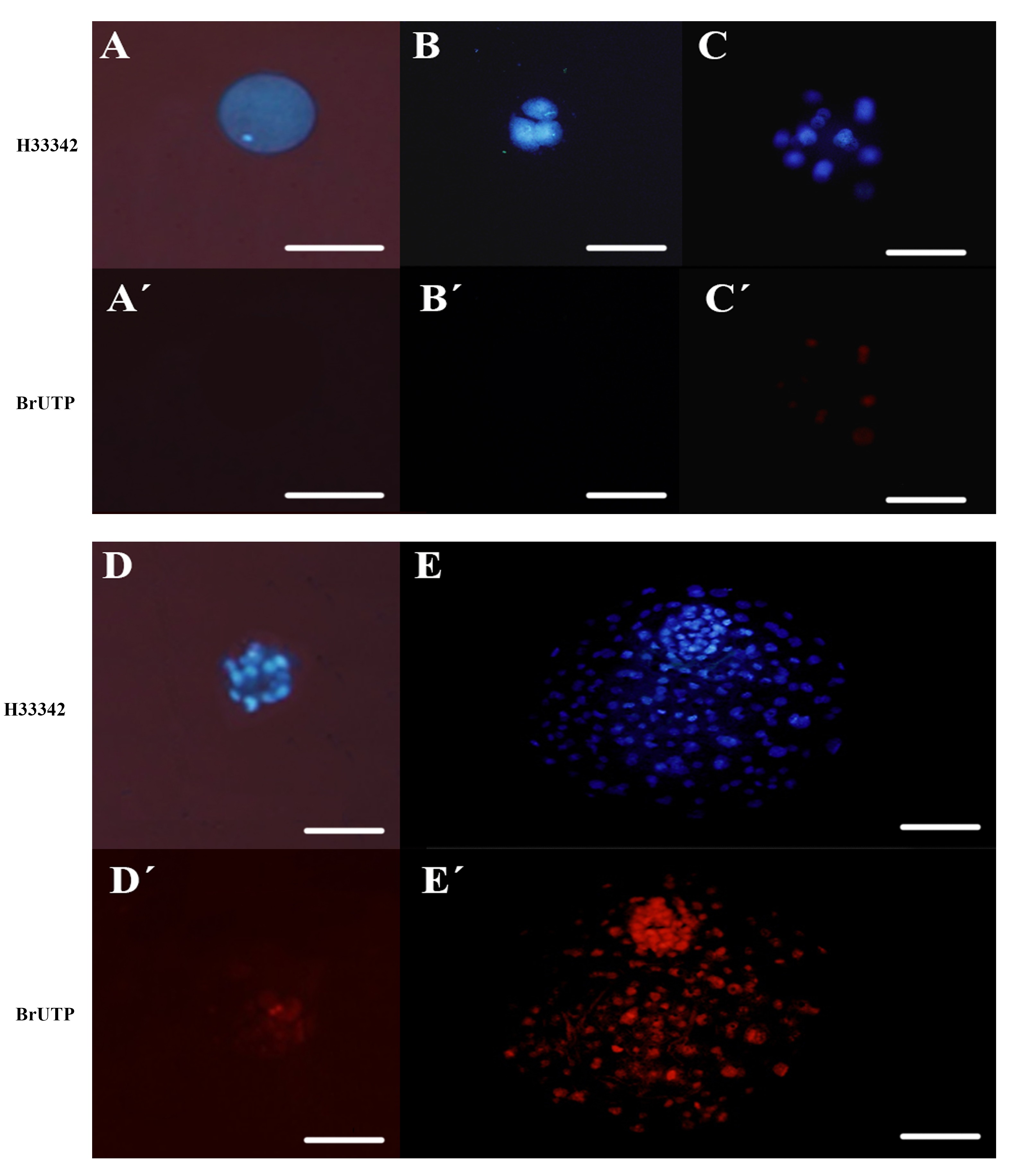

Supplement: Additional file 5: Figure S1. — Nascent RNA expression in CTR-NT and TSA-NT blastocysts. A-E’) Investigation of nascent RNA expression at different stages of cloned embryo development. Oocytes and blastocysts are considered as negative and positive controls based on RNA expression, respectively. No sign of mRNA expression was observed until the 8 (C&C’) -16 (D&D’) cell stage. Scale bar represents 100 μm. (JPEG 594 kb) [file 12864_2015_2264_MOESM5_ESM.jpeg]
