# Supplementary material for: Epigenetic modification with trichostatin A does not correct specific errors of somatic cell nuclear transfer at the transcriptomic level; highlighting the non-random nature of oocyte-mediated reprogramming errors
Source: BMC Genomics. 2016 Jan 4;17:16. doi: 10.1186/s12864-015-2264-z (PMC4698792; doi:10.1186/s12864-015-2264-z)
Supplement: Additional file 8: Table S7. — Real Time PCR gene expression analysis. Validation of microarray results by qRT-PCR quantification of the mRNAs in blastocysts developed in CTR-NT and TSA-NT groups compared to IVF. The analysis was done in five replicates of pools of ten blastocysts. ACTB was considered as housekeeping gene. (DOCX 17 kb) [file 12864_2015_2264_MOESM8_ESM.docx]

| Additional file 8: Table S7. **Real Time PCR gene expression analysis.** Validation of microarray results by reverse transcription–qRT-PCR quantification of the mRNA profiles in blastocysts developed in CTR-NT and TSA-NT groups compared to IVF. Analysis was done in five replicates of pools of ten blastocysts in each replicate. ACTB was considered as housekeeping gene. | | | | | |
| --- | --- | --- | --- | --- | --- |
|  | Microarray results | | qRT-PCR results | | |
| Gene | CTR-NT | TSA-NT |  | CTR-NT | TSA-NT |
| VEGFA | -1.0 | 1.0 |  | -1.0±0.4 | 1.3±0.2 |
| BCL2 | -1.0 | -1.1 |  | -1.0±0.0 | -1.0±0.3 |
| NANOG | -1.9* | -4.1* |  | -1.4±0.4 | -3.3±1.1* |
| POU5F1 | 1.2 | 1.6* |  | 1.5±0.2* | 1.0±0.3 |
| XIST | -1.7* | -1.6* |  | -0.6±0.7 | -0.4±0.1 |
| SOX2 | -1.1 | 1.0 |  | -1.0±0.2 | 1.1±0.3 |
| CDX2 | 1.2 | 1.3 |  | 1.5±0.5* | 1.4±0.3* |
| GSTM3 | -4.9* | -3.7* |  | -6.1±1.1* | -5.1±1.1* |
| HNF4a | 1.0 | 1.1 |  | 1.0±0.5 | 1.1±0.3 |
| C-MYC | 1.6* | 1.5* |  | 1.6±0.2* | 1.3±0.5* |
| BMPR1B | -1.1 | -1.3 |  | 1.3±0.4* | 1.9±0.7* |
| GATA4 | 1.6* | 2.0* |  | 2.2±0.2* | 2.3±1.1* |
| SMAD1 | 1.1 | 1.2 |  | -1.6±1.0* | -1.1±0.4 |
| CCNB1 | -1.9* | -2.4* |  | -2.3±1.1* | -3.4±0.8* |
| *Significantly different with IVF at P<0.05. | | | | | |
