# Supplementary material for: Epigenetic modification with trichostatin A does not correct specific errors of somatic cell nuclear transfer at the transcriptomic level; highlighting the non-random nature of oocyte-mediated reprogramming errors
Source: BMC Genomics. 2016 Jan 4;17:16. doi: 10.1186/s12864-015-2264-z (PMC4698792; doi:10.1186/s12864-015-2264-z)
Supplement: Additional file 9: Table S8. — qRT-PCR primers. Sequences (5′–3′) of reverse transcription qRT-PCR-specific primers for candidate genes expressed in bovine embryos. (DOCX 19 kb) [file 12864_2015_2264_MOESM9_ESM.docx]

| Additional file 9: Table S8. **qRT-PCR primers.** Sequences (5’-3’) of reverse transcription qRT-PCR-specific primers of candidate genes expressed in bovine embryos. | | |
| --- | --- | --- |
| **Gene** | **Name** | **Sequence** |
| **ACTB** | actin, beta | **F:** TCGCCCGAGTCCACACAG  **R:** ACCTCAACCCGCTCCCAAG |
| **VEGFA** | vascular endothelial growth factor A | **F:** TTCATTTTCAAGCCGTCCTC  **R:** CCTATGTGCTGGCTTTGGTG |
| **BCL2** | B-cell CLL/lymphoma 2 | **F:** AGCATCACGGAGGAGGTAGAC  **R:** CTGGATGAGGGGGTGTCTTC |
| **NANOG** | Nanog homeobox | **F:** CGTGTCCTTGCAAACGTCAT  **R:** CTGTCTCTCCTCTTCCCTCCTC |
| **POU5F1** | POU class 5 homeobox 1 | **F:** AGAAGGGCAAACGATCAAGC  **R:** AGGGAATGGGACCGAAGAGTT |
| **Sox2** | SRY (sex determining region Y)-box 2 | **F:** ATGGGCTCGGTGGTGA  **R:** CTCTGGTAGTGCTGGGA |
| **CDX2** | caudal type homeobox 2 | **F:** GCACCATCACCCTCACC  **R:** GGGCTTCCGCATCCACT |
| **GSTM3** | glutathione S-transferase mu 3 (brain) | **F:** GCGCTAAGGCACACAGGCGA  **R:** TGCGGGCGATGTAGCGCAAG |
| **HNF4a** | hepatocyte nuclear factor 4, alpha | **F:** CTGGAGACGACACGAG  **R:** TGGCTTTGGAGAAACGAG |
| **CTNNB1** | catenin (cadherin-associated protein), beta 1, 88kDa | **F:** AGTGGGTGGCATAGAGG  **R:** CACAGGTAGCCCGTAG |
| **C-myc** | C-myc myelocytomatosis viral oncogene | **F:** CAACACCCGAGCGACACC  **R:** GCCCGTATTTCCACTATCCG |
| **BMPR1B** | bone morphogenetic protein receptor, type IB | **F:** CCTGTTCGTCGTGTCTCAT  **R:** CTGGTGCTAAGGTTACTCC |
| **GATA4** | GATA binding protein 4 | **F:** TCCCCTTCGGGCTCAGTGC  **R:** GTTGCCAGGTAGCGAGTTTGC |
| **SMAD1** | SMAD family member 1 | **F:** TCACCATTCCTCGCTCCCT  **R:** AAACTCGCAGCATTCCAACG |
| **SFN** | stratifin | **F:** TCCGAGGCAGTCAGTTAG  **R:** CTTCACCCTTTTCCACCG |
| **STEAP2** | six transmembrane epithelial antigen of the prostate 2 | **F:** CCTACAGCCTCTGCTTACC  **R:** CCAGACTTCTTCCTCGTTCC |
| **UBD** | tubulin, delta 1 | **F:** ACGACCATCCACCTCACCC  **R:** CACCAGGCTCCACCAAAACC |
| **CXXC5** | CXXC finger 5 | **F:** TGTAGGAACCGAAAGACTG  **R:** CCGTCACTGAAACCACC |
| **ARHGEF6** | Rac/Cdc42 guanine nucleotide exchange factor (GEF) 6 | **F:** ACAGGGTCTTCAGATGGC  **R:** TTCCCCAGTCAGAGTTCC |
| **PPP1R15B** | protein phosphatase 1, regulatory (inhibitor) subunit 15B | F:CTCGTGGCAGGGATGTC  R:CTCCTCTAGGCTGTGGTA |
| **RAB9A** | RAB9A, member RAS oncogene family | **F:** GGAGATGGTGGTGTTGG  **R:** CCTCTGTAGAATGGTGTC |
| **SAP30L** | SAP30-like | **F:** GGAAGAAGGGGCTTGGTGG  **R:** CTTGCTGAAGGAGGCGTTGC |
| **HIST2H2B** | histone cluster 2, H2be | **F:** ATCCTTATCTTTTGTTCCCC  **R:** ATCCAACCTGTGTCTTCTG ) |
| **OXCT1** | 3-oxoacid CoA transferase 1 | **F:** GAGCCTCTTATTTCTCCAGT  **R:** TCCAGTTAGCCAGGTCAC |
| **LIX1L** | Lix1 homolog (mouse)-like | **F:** GCTTGAATAAGGGCTGTGA  **R:** AAGAGGAGTAAGGTGGTAG |
| **LAMA1** | laminin, alpha 1 | **F:** TCAGCACCAATGCGACC  **R:** TCCTAACAGAAGTCTCCAG |
| **ARPC1B** | actin related protein 2/3 complex, subunit 1B, 41kDa | **F:** TGGCACAGACCGCAACG  **R:** AGCAGATGGAGATGACACG |
| **MT1A** | metallothionein 1E | **F:** CAAATGGACCCGAACTG  **R:** TGGGCACACTTGGCAC |
| **SORLT** | sortilin-related receptor, L(DLR class) A repeats-containing | **F:** GGACGATGAGGAACAGAAGC  **R:** TCCATTAGCATAAGCAAAGAAGC |
| **XIST** | X (inactive)-specific transcript | **F:** TTGGCTTTTAGATTAATTTGATGAACAGCAT  **R:** CCCTTTAGACTAGGCCCATTTCATA |
